# Making Change: Attempts to Reduce or Stop Gambling in a General Population Sample of People Who Gamble

**DOI:** 10.3389/fpsyt.2022.892238

**Published:** 2022-08-17

**Authors:** David C. Hodgins, Robert J. Williams, Yale D. Belanger, Darren R. Christensen, Nady El-Guebaly, Daniel S. McGrath, Fiona Nicoll, Carrie A. Shaw, Rhys M. G. Stevens

**Affiliations:** ^1^Department of Psychology, University of Calgary, Calgary, AB, Canada; ^2^Faculty of Health Sciences, University of Lethbridge, Lethbridge, AB, Canada; ^3^Department of Political Science, University of Lethbridge, Lethbridge, AB, Canada; ^4^Department of Psychiatry, University of Calgary, Calgary, AB, Canada; ^5^Department of Political Science, University of Alberta, Edmonton, AB, Canada; ^6^Centre for Excellence in Responsible Gaming, University of Gibraltar, Gibraltar; ^7^University of Lethbridge, Lethbridge, AB, Canada

**Keywords:** gambling disorder, treatment-assisted change, natural recovery, problem gambling treatment, natural course of gambling disorder

## Abstract

**Objective:**

This study examined past year attempts to reduce or quit gambling among people who gamble generally and those with gambling problems specifically.

**Methods:**

Regular gamblers recruited from an online panel (*N* = 10,054) completed a survey of gambling, mental health and substance use comorbidity and attempts to reduce or quit gambling. The sample was weighted to match the gambling and demographic profile for the same subsample (i.e., past month gamblers) in a recent Canadian national survey.

**Results:**

5.7% reported that they tried to cutback or stop gambling in the past year. As predicted, individuals making a change attempt had greater levels of problem gambling severity and were more likely to have a gambling problem. Of individuals with problem gambling, 59.8% made a change attempt. Of those, 90.2% indicated that they did this primarily on their own, and 7.7% accessed formal or informal treatment. Most people attempting self- change indicated that this was a personal preference (55%) but about a third reported feeling too ashamed to seek help. Over a third (31%) reported that their attempt was successful. Of the small group of people accessing treatment, 39% described it as helpful.

**Conclusions:**

Whereas gambling treatment-seeking rates are low, rates of self-change attempts are high. The public health challenge is to promote self-change efforts among people beginning to experience gambling problems, facilitate success at self-change by providing accessible support for use of successful strategies, and provide seamless bridges to a range of other treatments when desired or required.

## Introduction

A frequently cited observation in the gambling field is that relatively few individuals with gambling problems seek treatment, with estimates ranging from 10 to 20% (1–4). This statistic promotes a call to action for greater range of treatment resources, with greater accessibility and a greater range of attractive options (1). Some studies have explored potential barriers to treatment-seeking (see Suurvali (2009) for a review). These studies, conducted across a variety of countries, consistently reveal that although perceived inaccessibility of treatment is not an uncommon barrier, the most frequently reported reasons are that people desire to change on their own and do not believe they require treatment (5).

Understanding peoples' attempts to change on their own can inform our efforts to design an effective treatment system (1). A line of “natural recovery” research has investigated self-recovery from gambling (6, 7). Most of these studies recruit convenience samples of individuals who have overcome gambling problems without treatment assistance. When compared to people who access treatment, self-recovery individuals tend to have slightly less severe gambling problem severity (7). These small sample studies provide rich detail about the recovery process for those individuals, including identifying the factors that precipitated their attempts at reducing gambling and the strategies that they used. One interesting observation is that the strategies that people describe using are very similar to those reported by individuals who accessed treatment (7), suggesting that processes of change maybe similar even if the routes differ.

One natural recovery study uniquely used a general population vs. a convenience sample to compare self- and treatment-assisted recovery from gambling disorder (8, 9). That study used a random digit dialing general population telephone survey to identify people who had experienced gambling problems, and those identified were then assessed in more detail about their current and past gambling. This study confirmed that treatment-assisted change in gambling was relatively rare, but it identified two types of self-recovery- a group that decided to reduce gambling and a group who “drifted out” of gambling because of other lifestyle changes without making a specific decision to address their gambling (9). This study confirms the importance of using representative samples as convenience designs did not sample the drifting out group, an understanding of which may be important in minimizing gambling-related harm through regulation and education. The current study meets this challenge by using a representative sample of people who gamble from an online panel. Individuals will be asked to describe any reductions in gambling, whether consciously initiated or a unprompted process.

## Current Study

The current study seeks to characterize the recovery process from gambling problems from a population perspective. The objectives of the current study were to:

(1) Determine the prevalence of attempts to reduce gambling in the past year among people who gamble generally, and those with gambling problems specifically, comparing the characteristics of individuals who attempted reduction to those not making an attempt. It is hypothesized that those making an attempt will have greater problem gambling severity.

(2) Compare individuals with gambling problems who attempted to make changes with the help of others relative to those attempting change on their own. It is hypothesized that individuals attempting to change on their own will have lower problem gambling severity and fewer comorbid mental issues.

(3) Describe the reasons that individuals making a change attempt on their own provide for not seeking support from others and their perceived success at self-change.

(4) Describe the range of formal treatment options accessed by individuals using outside help and their perceived helpfulness.

## Methods

### Participants

Data for this report are from the Alberta Gambling Research Institute's National Study on Gambling and Problem Gambling in Canada (https://www.ucalgary.ca/research/national-gambling-study/). As part of this project, a large sample of regular gamblers from across Canada was recruited from a pool of online panelists associated with the survey firm Leger360. This study was approved by the University of Lethbridge Human Ethics Review Board (Protocol#: 2018-063).

Repeated email notifications alerting members to a new survey were sent out until at least 1,400 completed surveys were obtained from each province or region (i.e., 1,400 each from the provinces of British Columbia, Alberta, Saskatchewan, Manitoba, Ontario, Quebec, and 1,400 from the four Atlantic provinces). The initial screening question identified active gamblers who reported they gambled at least once a month in the past year. Recruitment was open from August 15 to September 15, 2018, with a target of 10,000 active gamblers. Participants were invited to complete a follow-up survey 12 months afterward.

Participants were paid $10 CDN in addition to their standard panel participation incentives (monthly lottery draws) at both baseline and the follow-up survey. A total of 10,199 respondents self-identified as active gamblers according to the screening question but upon completion of the survey, 145 reported no gambling activity on any specific gambling format in the past 12 months. These respondents were excluded from the analysis leaving a final sample of 10,054.

Because online panels typically include more heavy gamblers than the general population, the sample was weighted to match the gambling behavior and demographic profile for the same subsample (i.e., past month gamblers) in the national Canadian Community Health Survey 2018 Gambling Module (10). Weights were created via raking with following variables: number of types of gambling, Problem Gambling Severity Index (11) category, provincial population, gender, education, and age. Weights were then winsorized to a maximum value of six times the average weight to minimize mean squared error.

### Survey Instrument

Participants completed a self-administered online questionnaire covering demographic questions and a range of topics related to gambling and substance use and mental health. For the current study our primary interest was data pertaining to gambling participation, gambling-related harm, comorbid mental health, recent life events, and a set of variables describing attempts to reduce or quit gambling and use of treatment. Information on gambling involvement was collected using the Gambling Participation Instrument (12), a reliable and valid tool for assessing the intensity and breadth of gambling engagement across the most common formats available to Canadians. Gambling was summarized in terms of typical monthly expenditure and frequency. The total expenditure on all forms of gambling was calculated by summing the expenditures for the individual gambling formats. Frequency was also assessed separately for each gambling format and summed. The original 7-point categorical scale was converted to a quantitative scale to estimate mean number of gambling days each month (1–5 times/year = 0.25 days; 6–11 times/year = 0.5 days; 1 time/month = 1 day; 2–3 times/month = 2.5 days; once per week = 4 days; 2–6 times/week = 16 days; daily = 30 days).

Problem gambling severity was assessed using the Problem Gambling Severity Index (PGSI), a nine-item scale that assesses consequences and behavioral symptoms of problem gambling in the past 12 months (11). The PGSI has good internal consistency (α = 0.95 within the current sample) and test-retest reliability (13). We used both the total PGSI score (range 0 to 27) and dichotomous cut-off of 5 for identifying problem gamblers (13, 14). Gambling fallacies were assessed with the 10-item Gambling Fallacies Questionnaire (GFQ), which yields a summed total score. A lower score indicates greater endorsement of gambling fallacies (15). Comorbidities assessed included past year use of tobacco, alcohol, cannabis, and non-medical use of other drugs; past year substance use disorder (assessed using DSM-5 criteria (16); past year behavioral addictions (excessive and problematic engagement in over eating, sex or pornography, exercise, shopping, social media, video games, Internet, other); history of child abuse or neglect; number of significant negative past year life events (39 item checklist, validated in previous research (17); and past year post-traumatic stress, generalized anxiety, panic disorder, and major depression (each assessed using DSM-5 criteria and summarized in a composite variable of any past year mental health disorder). Family history of gambling problems was assessed with an item asking whether anyone in their immediate family ever had a gambling problem (i.e., had difficulty controlling their gambling to the extent that it caused significant problems), followed by questions determining which relatives and whether they biological or not.

All participants were asked whether they had tried to cut down or stop gambling in the past year. Individuals who indicated ‘yes', and who also had a PGSI score of 5 or greater were asked a series of questions designed to elicit information whether they tried this primarily on their own or with the help of others. Individuals who attempted to change on their own were asked why they did not seek external help, including four checklist options derived from previous research. People who sought the help of others were asked what kind of help they received (see **Table 3** for the 12 checklist options), whether their change attempt was successful (not at all, somewhat, quite, very successful).

### Analysis

To address objectives 1 and 2, groups are described using frequencies, means, and standard deviations. We report group comparisons using percentages and Cohen's D effect sizes. For categorical variables, the odds ratio was converted to Cohen's d (18). Standard effect size interpretations were adopted (*d* = 0.2, small; *d* = 0.5 medium; *d* = 0.80 large) (19). The analyses reported in this manuscript were not pre-registered. All analysis were conducted using SPPS, V28. Other reports not focusing on the variables and relations examined in the present article have been or will be published (https://www.ucalgary.ca/research/national-gambling-study/). The dataset will become publicly available through Gambling Research Exchange Ontario in 2023.

## Results

Of the total sample, 1,598 individuals (15.9%, 5.7% of the weighted sample) reported that they tried to cutback or stop gambling in the past year. See Supplementary Figure 1 for a description of cell sizes. As predicted, individuals making a change attempt had greater problem gambling severity scores (PGSI *M* = 7.4, *SD* = 6.6) than those not reporting a change attempt (*M* = 1.0, *SD* = 3.0), *t*_(10052)_ = 61.1, *p* < 0.0001, *d* = 3.8. They also were more likely to have a gambling problem (PGSI 5+; 56.9% vs. 7.0%), χ^2^(1, *N* = 10,054) = 2644.0, *p* < 0.00001.

Limiting the sample to 1,497 individuals with problem gambling (PGSI 5+), 909 (60.7, 59.8% weighted) made a change attempt. By comparison, only 8.1% (4.7% weighted) of non-problem gamblers reported an attempt. Of those with a gambling problem, 796 (87.6, 90.2% weighted) indicated that they did this primarily on their own, and 113 indicated that did with the help of others (14.4, 9.8%, weighted) including 93 who accessed formal or informal treatment (10.2, 7.7% weighted).

Table 1 compares those making a change attempt to those not reporting a change attempt on a range of demographic, mental health, substance use and gambling characteristics, including effect sizes. Results are displayed for the entire sample and for the subsample of individuals with problem gambling. For the total sample, generally demographic variables showed a small size association. Younger people, males, not married, unemployed, and less educated were slightly more likely to report a change attempt. Comorbidity variables showed small to medium effect size with greater comorbidity associated with greater likelihood of an attempt. Presence of another behavioral addiction and number of negative life events showed the largest effect sizes. Gambling engagement variables, except for gambling losses, generally showed a medium effect size with greater gambling engagement and more endorsement of gambling fallacies associated with attempts to change. Indicators of gambling problem severity showed the largest effect size, consistent with our hypothesis.

**Table 1:** Who makes change attempts among people who gamble and people with gambling problems?.

		**Total Sample**			**Problem Gambling Sample**	
	**No**	**Yes**	**ES**	**No**	**Yes**	**ES**
*n*	8,456	1,598		588	909	
Age (M, SD)	52.8(15.2)	46.9(17.1)	0.37	38.8 (14.1)	40.3 (14.6)	0.10
Gender (%) male	54.3	65.7	0.26	72.3	67.6	0.12
female	45.6	34.3		27.7	32.4	
other	0.1	0		0	0	
**Ethnic/cultural origins (% yes)**						
Western and Northern European	62.2	52.5	0.22	38.0	43.3	0.12
Eastern European	9.8	10.0	0.02	12.5	11.2	0.07
Indigenous North American	4.3	5.8	0.18	6.9	6.5	0.04
Southern European	4.3	3.9	0.06	2.8	4.7	0.30
Chinese	2.4	2.8	0.09	4.2	2.8	0.23
African	1.1	7.0	1.05	2.8	8.5	0.64
South Asian	1.2	2.3	0.37	8.3	3.7	0.47
Southeast Asian	1.0	2.6	0.56	2.8	1.9	0.22
Latin American	0.7	2.6	0.72	1.4	4.7	0.69
Middle Eastern and Arab	0.4	0.7	0.34	2.8	0.9	0.62
East Asian	0.5	0.5	0.00	1.4	0.9	0.22
Central and Northern Asian	0.2	0.5	0.53	1.4	0.9	0.22
Marital status (% common-law/married)	64.5	49.7	0.34	46.0	37.0	0.20
Employed full-time (% yes)	44.7	36.6	0.19	52.4	40.2	0.27
Education (% post high school)	65.6	56.7	0.20	59.4	49.6	0.22
Alcohol (more than weekly)	36.4	27.6	0.23	37.5	30.0	0.18
Substance use disorder (% yes)	3.8	9.2	0.52	23.2	22.5	0.02
Cannabis (% yes)	20.6	31.2	0.31	54.7	46.3	0.18
Tobacco (% yes)	30.3	41.8	0.28	69.5	59.8	0.24
Behavioral addiction (past year)	9.8	27.8	0.70	34.0	43.2	0.22
Mental health disorder (% yes)	21.7	34.1	0.34	40.1	55.5	0.34
Negative life events (M, SD)	1.3(2.0)	2.1(2.6)	0.61	2.6 (3.3)	3.3 (3.2)	0.20
Child abuse/neglect (% yes)	17.9	23.6	0.19	31.1	32.7	0.04
Number of gambling types (*M, SD)*	2.1(1.1)	2.8(1.5)	0.57	3.9 (1.9)	3.9 (2.0)	0.01
Online gambling (% yes)	14.5	27.7	0.45	49.8	49.9	0.00
Gambling fallacies (M, SD)	6.7(1.5)	5.9(1.9)	0.47	4.9(2.1)	5.0(2.3)	0.03
PGSI (M, SD)	0.8	18.7	1.88	9.8 (4.2)	11.7 (5.9)	0.35
Family history problem gambling (%)	7.1	16.4	0.52	22.5	26.2	0.11
Gambling frequency/month (M, SD)	5.3(2.9)	7.4(4.5)	0.55	9.8 (4.2)	10.6 (6.8)	0.13
Gambling losses/month $ (M, SD)	223.3(1047.5)	636.6(2602.3)	0.21	1084.95(3402.88)	1584.76(4740.28)	0.12

*ES, effect size [Cohen's d for continuous and odds ratio converted to Cohen's d for categorical variables, Chinn (18)]; PGSI, Problem Gambling Severity Index; PPGM, Problem and Pathological Gambling Measure. Weighted data*.

For the subsample of individuals showing evidence of problem gambling, effect sizes were generally small or lower. Only frequency of gambling disorder diagnosis fell in the medium to large range. The next largest difference was presence of mental health disorder. It is notable that several the substance use indicators, specifically higher cannabis, tobacco, and substance use disorder, predicted a change attempt in the total sample but had the opposite association in the gambling problem sample -among people with gambling problems presence of those comorbidities was less likely among people making a change attempt.

Table 2 compares people with gambling problems who change on their own vs. those seeking outside help (objective 2). The small subgroup of people who indicated that they sought outside help but only from family and friends (*n* = 20) are omitted. As predicted, greater gambling problem severity was strongly associated with treatment seeking. Other gambling variables showed much more modest albeit still moderate to large effect sizes. Demographic characteristics showed small effect sizes and the strength of association was variable for comorbidity variables.

**Table 2:** Individuals with gambling problems attempting self- change or treatment-assisted change.

	**Self**	**Treatment**	**ES**
Age (M, SD)	40.6(14.6)	37.2(13.2)	0.25
Gender (%) Male	68.1	63.5	0.11
Female	31.9	36.5	
Other	0	0	
**Ethnic/cultural origins (% yes)**			
Western and Northern European	42.2	49.6	0.16
Eastern European	11.5	10.8	0.04
Indigenous North American	6.1	11.3	0.37
Southern European	4.5	7.3	0.28
Chinese	2.6	3.4	0.16
African	9.1	2.9	0.67
South Asian	4.9	3.2	0.13
Southeast Asian	1.7	4.2	0.51
Latin American	4.6	1.9	0.52
Middle Eastern and Arab	0.7	3.0	0.78
East Asian	1.3	1.4	0.03
Central and Northern Asian	0.4	1.4	0.67
Marital status (% common-law/married)	35.8	56.9	0.48
Employed full-time (% yes)	40.4	35.4	0.12
Education (% post high school)	49.3	54.7	0.12
Alcohol (more than weekly)	29.2	39.4	0.25
Substance use disorder (% yes)	21.6	35.8	0.39
Cannabis (% yes)	44.7	60.8	0.36
Tobacco (% yes)	58.1	85.2	0.79
Behavioral addiction (past year)	42.1	51.6	0.21
Mental health disorder (% yes)	54.0	72.1	0.44
Negative life events *(M, SD)*	3.1(3.1)	5.3(3.7)	0.66
Child abuse/neglect (% yes)	31.5	42.3	0.26
Number of gambling types (*M, SD)*	3.8(1.9)	5.5(2.1)	0.87
Online gambling (%)	47.7	77.2	0.72
Gambling fallacies (M, SD)	5.0(2.2)	4.5(2.5)	0.21
PGSI total score (M, SD)	11.4(5.8)	14.01(5.3)	5.71
Family history problem gambling (%)	23.7	55.2	0.76
Gambling frequency (M, SD)	10.3(6.7)	14.0(7.8)	0.51
Gambling losses $ (M, SD)	1568.4(4784.2)	1571.6(3152.1)	0.0008

*ES, effect size [Cohen's d for continuous and odds ratio converted to Cohen's d for categorical variables, Chinn (18)]. PGSI, Problem Gambling Severity Index; PPGM, Problem and Pathological Gambling Measure. Weighted data*.

Objective 3 concerned the reasons for not seeking help given by individuals making a change attempt on their own. These individuals were presented four possible reasons that they did not seek external support and could endorse multiple options. The most frequently endorsed reason was that “I did not believe I needed help” (55%) followed by feeling “too ashamed to seek help” (31%). The final two reasons were less frequently endorsed – I was unaware of where to get help (18%), and “I did not believe that treatment would work for me” (16%). These individuals were also asked to rate the success their self-change attempt. Only 13.4% indicated “not at all” with 49.0% indicating somewhat, 22.1% quite, and 15.5% very successful.

The fourth objective queried the range of formal treatment options accessed and how individuals became aware of these services. Individuals who sought treatment were provided with a list of 11 options plus “other.” Individuals generally sought out multiple options, endorsing a mean of 2.0 (SD = 1.4). Table 3 displays the frequency of use. These individuals were asked to rate the helpfulness of the treatments provided. Most frequently the help was rated as somewhat helpful (44.3%) with 16.0% indicating not at all, 20.9% quite helpful, and 18.8% very helpful.

**Table 3:** Formal treatments accessed (*N* = 909).

**Treatment**	**%**
Self-help materials	22.0
Self-exclusion program	15.7
Psychologist, psychiatrist, counselor – in person	10.4
Primary care physician	10.1
Online or telephone service	9.6
Gamblers anonymous or other support group	8.8
Clergy	5.2
Medication	4.6
Residential program	2.0
Other	17.7

*Weighted data*.

## Discussion

Treatment-seeking was a relatively rare event in this sample. The proportion of people showing evidence of gambling problems that sought treatment was low (14.4% in the unweighted sample, 9.8% in the weighted sample) and almost identical with earlier observations in Canada and elsewhere. Consistent with earlier research as well, people who sought treatment tended to have more serious gambling problems and greater overall gambling engagement. An exception is amount of gambling losses, which was unassociated with whether individuals sought treatment. This may relate to the large variability of losses in the sample (from none to $111,000). The demographic variable that was most strongly associated with greater treatment-seeking was being married or in a common-law relationship. Of interest, gender was not strongly predictive of treatment seeking, although, as expected, males generally were more prevalent in the problem gambling subgroup than females. Presence of comorbidities was higher in the treatment-seeking group, but only tobacco smoking showed a strong effect. Over 85% of individuals seeking treatment were smokers, which reinforces the importance of addressing this issue in gambling interventions (20) and screening for gambling problems in smoking cessation programs. Similarly, mental health and substance use disorders were relatively prevalent among treatment-seekers (72% and 36%), which have implications for gambling and mental health interventions (21, 22).

Despite the low rates of treatment-seeking, this sample demonstrated that lots of change attempts are occurring. Among people with gambling problems, 60% reported a change attempt in the past year. This high figure is a cause for optimism as it indicated good self-awareness of issues and willingness to address them. Although some of these individuals chose to access formal or informal treatment, over 90% elected to attempt to reduce or quit on their own. Whether this is a less effective way than formal treatment to address their problem is unclear. However, when asked about their success, about half of these self-changers indicated that they had been “somewhat successful.” Although only 13% indicated no success, only 38% indicated being quite or very successful. These findings underscore the importance of work that seeks to understand how people are successful at self-change and how to promote it (1, 23, 24).

Most people electing to change without help indicated that they did not believe they needed treatment. However, many reported feeling too ashamed about their situation, lacking information about treatment options, and lack of confidence in treatment. These findings show the importance of ongoing promotion of self-help materials, treatment availability, optimistic messages about treatment success, and, importantly, stigma reduction. Within a stepped care model framework, self-change, if unsuccessful, is best followed by more structured help (1). Interestingly, those who accessed treatment most frequently indicated that a family member or friend obtained the information for them. This reinforces the importance that awareness messages be directed not only at the individual who is gambling, but also concerned family and friends.

We asked individuals to rate the helpfulness of the treatments they accessed (overall, not each treatment individual). These proportions paralleled people's ratings of the success of self-change efforts. Most frequently the help was rated as somewhat helpful (44.3%) with 16.0% indicating not at all, 20.9% quite helpful, and 18.8% very helpful. It seems like treatment is generally helpful and is positively experienced by most people.

The value of this study is that it provides a population perspective on recovery from gambling problems, compared to smaller studies of convenience samples. The disadvantage is that important and rich detail that would have been helpful, was not obtained in this online survey. For example, we did not obtain a full picture of the treatment experiences of individuals, whether individuals' self change attempts were conscious or represented “drifting out” or the details of how and why they made a change attempt without treatment. We also do not have information about how people defined success or being somewhat successful and how this was associated with their concept of recovery. We do not know whether their goal was total abstinence, abstinence from specific types of gambling or reduced gambling, which can be important in outcomes (25). In addition, how people think about successful change in the context of feeling shame and stigma is unclear. Moreover, there was a large subsample of respondents who did not meet criteria for problem gambling, but nonetheless proactively indicated an attempt to reduce their gambling. This subgroup should be examined in future research including further study of their motivations and change strategies. Our sampling strategy that recruited people who typically gamble monthly complicated understanding this subgroup.

This study relied on self-reported data from an online survey sample so it will include some error measurement. Some important concepts were measured with unvalidated items from previous surveys (e.g., based on DSM criteria). The large, well-characterized diverse sample compensates somewhat but specific estimates need to be interpreted cautiously.

In conclusion, the results of this study align well-with previous research with smaller samples of convenience, which is reassuring. The findings validate the observation that treatment-seeking rates are low, but they also emphasize that rates of self-change attempts are high. The public health challenge is threefold: (1) promote self-change efforts among people beginning to experience struggles with gambling; (2) facilitate success at self-change by providing accessible and accurate advice and support for use of successful strategies; and (3) provide seamless bridges to a range of other treatments when desired or required.

## Data Availability Statement

The raw data supporting the conclusions of this article will be made available by the authors, without undue reservation.

## Ethics Statement

The studies involving human participants were reviewed and approved by University of Lethbridge Ethics Review Board. The patients/participants provided their written informed consent to participate in this study.

## Author Contributions

CS managed the project data collection and database development. CS and RW consulted on statistical analyses. DH conducted analyses and wrote the first draft of the paper. All authors were involved in the project conceptualization and reviewing the submitted manuscript.

## Funding

This project was funded by the Alberta Gambling Research Institute.

## Conflict of Interest

The authors declare that the research was conducted in the absence of any commercial or financial relationships that could be construed as a potential conflict of interest.

## Publisher's Note

All claims expressed in this article are solely those of the authors and do not necessarily represent those of their affiliated organizations, or those of the publisher, the editors and the reviewers. Any product that may be evaluated in this article, or claim that may be made by its manufacturer, is not guaranteed or endorsed by the publisher.
