# Supplementary material for: Making Change: Attempts to Reduce or Stop Gambling in a General Population Sample of People Who Gamble
Source: Front Psychiatry. 2022 Aug 17;13:892238. doi: 10.3389/fpsyt.2022.892238 (PMC9428338; doi:10.3389/fpsyt.2022.892238)
Supplement: Supplementary file 1 [file Table_1.DOCX]

**Supplementary Figure 1.** Recruitment and categorization of respondents.
